# Pvf/Pvr signaling relieves white spot syndrome virus-induced lipid consumption to inhibit viral infection in shrimp

**DOI:** 10.1128/jvi.00182-26

**Published:** 2026-04-29

**Authors:** Zi-Hua Cheng, Ping-Ping Liu, Ze-Xuan Fan, Meng Zhang, Shu-Dian Zhang, Yuan-Ning Li, Dong-Wei Kang, Xian-Wei Wang

**Affiliations:** 1 School of Life Sciences, Shandong University 520252, Qingdao, Shandong, China; 2 State Key Laboratory of Microbial Technology, Shandong University 520252, Qingdao, Shandong, China; 3 Institute of Marine Science and Technology, Shandong University 520252, Qingdao, Shandong, China; 4 Department of Medicinal Chemistry, School of Pharmaceutical Sciences, Cheeloo College of Medicine, Shandong University 66555, Jinan, Shandong, China; Wageningen University & Research, Wageningen, Netherlands

**Keywords:** Pvf/Pvr signaling, lipid metabolism, shrimp, white spot syndrome virus

## Abstract

Platelet-derived growth factor (PDGF) and vascular endothelial growth factor (VEGF)-related factors (Pvfs) are highly conserved and widespread growth factors whose signaling is mediated by the PDGF/VEGF-related receptor (Pvr). Pvf/Pvr signaling is involved in the regulation of development, immunity, and metabolism of organisms and can be a key therapeutic target for a variety of clinical diseases. However, the functions and mechanisms of Pvf/Pvr signaling in viral infections are largely unknown. In the present study, we found that Pvf/Pvr family members, MjPvf2/MjPvr3, inhibit white spot syndrome virus (WSSV) infection by regulating lipid metabolism in the kuruma shrimp (*Marsupenaeus japonicus*). WSSV infection caused a pronounced reduction in triglyceride content, whereas the activation of MjPvf2/MjPvr3 signaling relieved lipid consumption and suppressed viral infection. Mechanistically, WSSV infection enhanced MjPvf2/MjPvr3 signaling, leading to the activation of extracellular signal-regulated kinase (ERK) and sterol regulatory element-binding protein (SREBP), and subsequent induction of hydroxysteroid dehydrogenase (*MjHSD*) expression. MjHSD promoted lipid synthesis and restored lipid levels, thereby restricting viral replication. Importantly, pharmacological inhibition of lipid synthesis eliminated the antiviral effect of MjPvf2/MjPvr3 signaling. In conclusion, this study revealed the significance and mechanism of Pvf/Pvr signaling in antiviral immunity, providing novel insights into WSSV prevention and control in shrimp aquaculture.

**IMPORTANCE:**

Viral infection affects host lipid metabolism. This process depletes the host’s lipids to support viral replication. Pvf/Pvr signaling is widely involved in host physiological processes, but its role in viral infection has not been clarified. We demonstrate that Pvf/Pvr signaling exerts an antiviral effect by relieving the lipid consumption caused by white spot syndrome virus (WSSV) infection in shrimp. This study provides new insights into the effects of Pvf/Pvr signaling on antiviral immunity and highlights the complexity of lipid metabolism under WSSV infection.

## INTRODUCTION

Platelet-derived growth factor (PDGF) was originally discovered as a serum factor that stimulates the proliferation of arterial smooth muscle cells (1). The PDGF family includes PDGF, vascular endothelial growth factor (VEGF), and placental growth factor, whose members are produced by a variety of cells, and their receptors are receptor tyrosine kinases (2). In mammals, PDGF/VEGF signaling has been extensively studied and is known to regulate physiological activities related to angiogenesis (3) and has been used as a drug target for diseases including atherosclerosis, cancer, fibrotic diseases, and neurological disorders (4). In *Drosophila*, this protein family regulates several physiological processes. VEGF signaling controls the development and migration of blood cells, and this movement is blocked when its receptor is mutated (5). Moreover, *Drosophila* intestinal commensal bacteria-induced NF-κB signaling and viruses-triggered Cdk9-mediated transcriptional activation synergistically induce PDGF/VEGF-related factor 2 (Pvf2) expression. Secreted Pvf2 subsequently binds to the PDGF/VEGF-related receptor (Pvr) on intestinal epithelial cells, mediating extracellular signal-regulated kinase (ERK) phosphorylation and activation of downstream antiviral immunity (6). In addition, Pvf3 produced by blood cells acts on Pvr in fat body cells, thereby regulating cell size and triglyceride (TG) storage (7). PDGF/VEGF-related proteins have been identified and characterized in various crustaceans (8–10). However, the functions of this family have not yet been fully investigated in shrimp.

Virus-lipid interactions influence the entire process of viral infection, from host invasion to replication, assembly, and release. Many viruses affect host lipid metabolism by activating lipid-related signaling pathways and inducing lipid-related gene expression, making lipid metabolism a key indicator of viral infection outcomes (11). Hepatitis C virus (HCV) infection is closely associated with lipid metabolic disorders, characterized by reduced apolipoprotein B, low-density lipoprotein cholesterol, and total serum cholesterol levels, accompanied by increased hepatic steatosis (12). Dengue virus (DENV) induces autophagy-mediated TG breakdown to release free fatty acids, which undergo mitochondrial β-oxidation to generate ATP and promote viral replication (13). In parallel, DENV nonstructural protein 3 recruits and activates fatty acid synthase at the viral replication site to promote fatty acid synthesis (14). Human cytomegalovirus (HCMV) infection broadly alters cellular metabolic processes, including fatty acid synthesis, and inhibition of this pathway suppresses the replication of HCMV and influenza A virus, highlighting the requirement of fatty acid synthesis for enveloped virus replication (15, 16). In addition to fatty acids, cholesterol and complex lipids also contribute to viral infection. West Nile virus induces host cholesterol biosynthesis and redistributes cholesterol to viral replication sites, facilitating viral replication and evasion of the host antiviral response (17). Gangliosides (GM) function as viral receptors that mediate intracellular trafficking, with GD1a/GT1b recognizing polyomavirus and GM1 binding simian virus 40 (18). Omega-3 polyunsaturated fatty acids produce lipid-mediated protective protein D1, which inhibits influenza A virus replication by blocking viral RNA export and promotes host survival (19). Triglyceride synthase diacylglycerol acyltransferase-1 binds to the HCV core protein and localizes to lipid droplets to facilitate viral particle assembly (20). In shrimp, Mindin activates autophagy to deplete lipids and promote white spot syndrome virus (WSSV) infection, suggesting that lipid metabolism also influences WSSV infection (21).

Shrimp aquaculture is an economically important industry in many coastal areas but has long been challenged by infectious diseases. WSSV remains the most devastating viral pathogen affecting shrimp farming. Despite increasing evidence that lipid metabolism critically influences viral infection, the interactions between host lipid regulation and WSSV infection remain poorly understood. Given the regulatory roles of Pvfs in host physiology, we hypothesized that Pvf signaling may participate in virus-lipid interactions. In this study, we investigated the role of the Pvf family in WSSV infection in the kuruma shrimp, *Marsupenaeus japonicus*. We found that MjPvf2/MjPvr3 signaling inhibits WSSV infection in shrimp. Mechanistically, MjPvf2/MjPvr3 signaling activates the downstream ERK/sterol regulatory element-binding protein (SREBP) pathway, inducing the expression of *MjHSD* and thereby regulating lipid levels. Importantly, the antiviral effect of MjPvf2/MjPvr3 signaling depends on enhanced lipid synthesis, which counteracts WSSV-induced lipid consumption. Therefore, our findings revealed a lipid metabolism-dependent antiviral mechanism in shrimp and provide new insights into virus-lipid interactions relevant to WSSV control in shrimp aquaculture.

## RESULTS

### MjPvf2 responds to and inhibits WSSV infection

**Fig 1:**
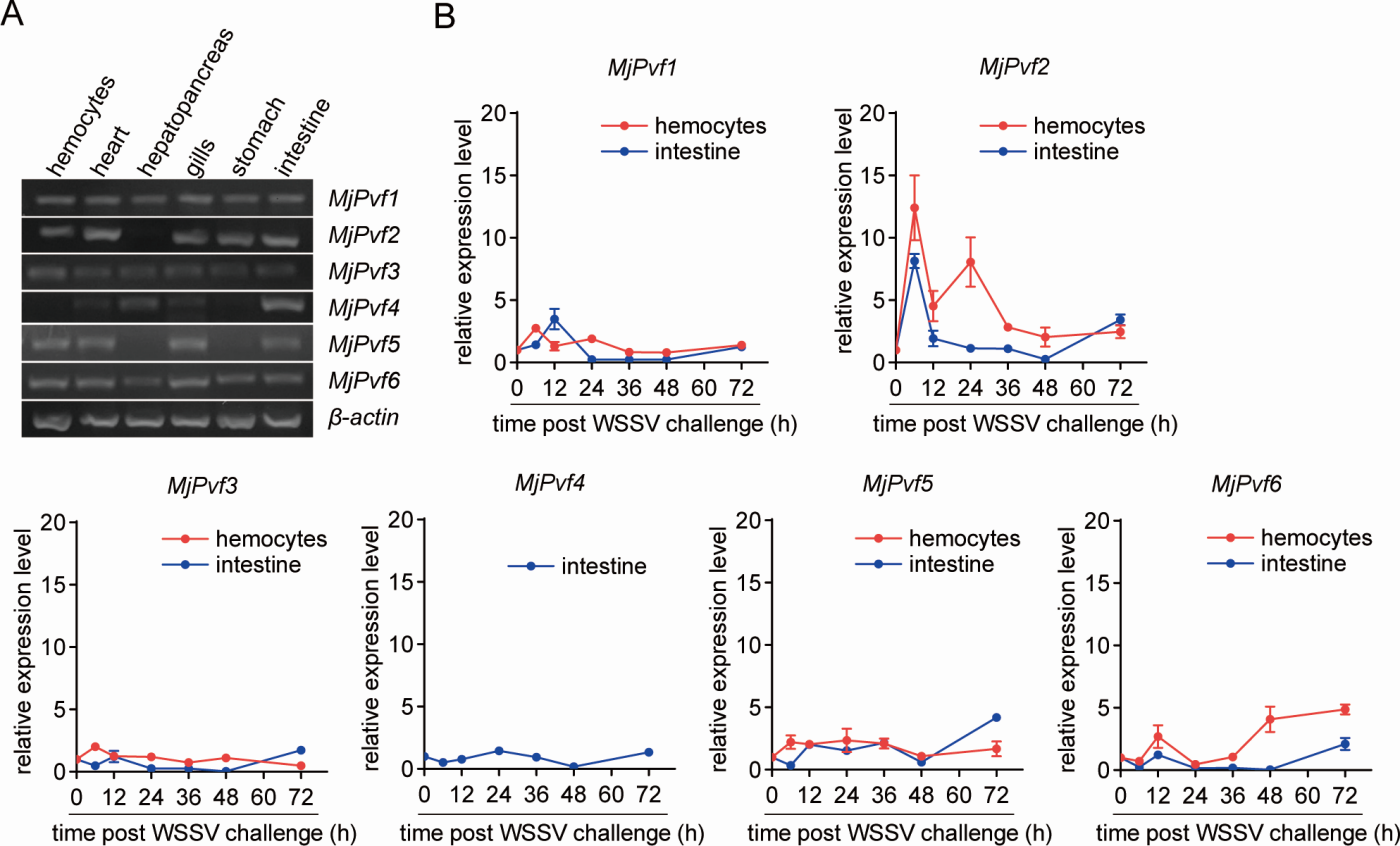
MjPvf2 expression responds to WSSV infection. (**A**) Tissue distribution of *MjPvf*s mRNA. Total RNA was extracted from healthy shrimp. Gene expression was detected using RT-PCR with *β-actin* as an internal reference. Each set of samples contained at least five shrimp. Data represent the results of two independent experiments. (**B**) Expression pattern of *MjPvf*s in shrimp hemocytes and intestine after WSSV infection. Total RNA was extracted from shrimp hemocytes at the corresponding time points after WSSV infection. qRT-PCR was used to detect gene expression, using *β-actin* as an internal reference. Expression levels at each time point are normalized to those of the control group. The results are shown as the mean ± SD. All samples were obtained from at least five shrimps, and the experiment was repeated three times.

The tissue distribution of *MjPvf* family members at the transcriptional level was analyzed. Six members were identified in *M. japonicus* and designated MjPvf1-6 (Fig. 1A). *MjPvf1*, *MjPvf3,* and *MjPvf6* exhibited ubiquitous expression across all examined tissues; *MjPvf2* showed pan-tissue expression except in hepatopancreas, while *MjPvf4* was universally expressed, excluding hemocytes and stomach; and *MjPvf5* was also universally expressed, excluding hepatopancreas and stomach. To investigate whether *MjPvf*s are involved in WSSV infection, their expression profiles were examined in hemocytes and intestines at different time points after WSSV injection. As shown in Fig. 1B, among all family members, *MjPvf2* exhibited a robust response to WSSV infection in both hemocytes and intestine, suggesting that *MjPvf2* might be involved in viral infection.

**Fig 2:**
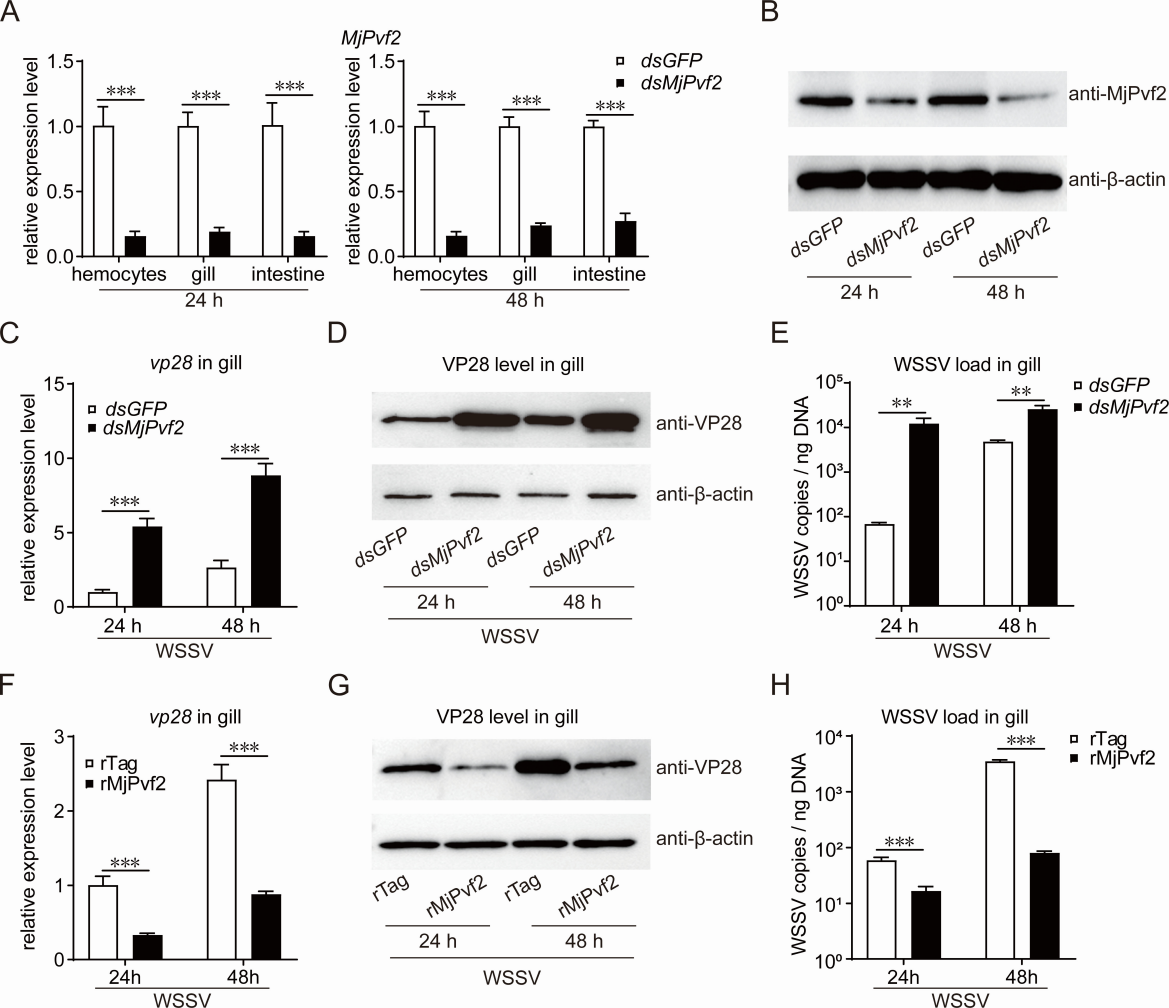
MjPvf2 inhibits WSSV infection. (**A and B**) RNAi efficiency of *MjPvf2*. Total RNA was extracted from different tissues 24 h and 48 h after dsRNA injection. qRT-PCR was used to detect the RNAi efficiency (**A**). Total protein in the intestine was extracted and detected by western blotting 24 h and 48 h after dsRNA injection (**B**). (**C–E**) Effect of *MjPvf2* knockdown on WSSV infection. Shrimp were infected with WSSV (5 × 10^5^ copies) 24 h after dsRNA injection. Total RNA, protein, and genomic DNA in gill were extracted 24 and 48 h post-infection. qRT-PCR was used to detect WSSV *vp28* transcript levels (**C**). Western blotting was used to detect WSSV VP28 translation levels, using β-actin as an internal reference (**D**). qPCR was used to detect the WSSV viral loads (**E**). (**F–H**) Effect of rMjPvf2 administration *in vivo* on WSSV infection. Shrimp were injected with rMjPvf2 or rTag (3 µg) while infected with WSSV (5 × 10^5^ copies). Total RNA, protein, and genomic DNA in gill were extracted 24 and 48 h post-infection. WSSV *vp28* transcript levels (**F**), VP28 translation levels (**G**), and viral loads (**H**) were detected. Data are the mean ± SD of three independent replicates. Statistical analysis was performed using the Student’s *t-*test. **P < 0.01, ***P < 0.001, and ns, not significant. All samples were obtained from at least five shrimp, and the experiment was repeated three times.

To investigate the function of MjPvf2 during WSSV infection, RNA interference (RNAi) was performed to knock down *MjPvf2* expression. Application of *MjPvf2*-specific dsRNA efficiently reduced MjPvf2 expression at both the mRNA (Fig. 2A) and protein levels (Fig. 2B). Following WSSV infection, *MjPvf2* knockdown resulted in a significant increase in the transcription of *vp28*, which encodes the most abundant envelope protein of WSSV (Fig. 2C). Consistently, VP28 levels (Fig. 2D) and the WSSV load (Fig. 2E) were markedly elevated in *MjPvf2*-silenced shrimp. To further validate the antiviral role of MjPvf2, recombinant MjPvf2 protein (rMjPvf2) was injected into shrimp hemocoels to mimic enhanced MjPvf2 signaling. The results showed that rMjPvf2 significantly inhibited WSSV *vp28* transcription (Fig. 2F), VP28 levels (Fig. 2G), and the viral load in tissues (Fig. 2H). These results suggested that MjPvf2 responds to WSSV infection and functions as an antiviral factor that restricts WSSV replication in shrimp.

### MjPvf2 interacts with MjPvr3 to inhibit viral infection

**Fig 3:**
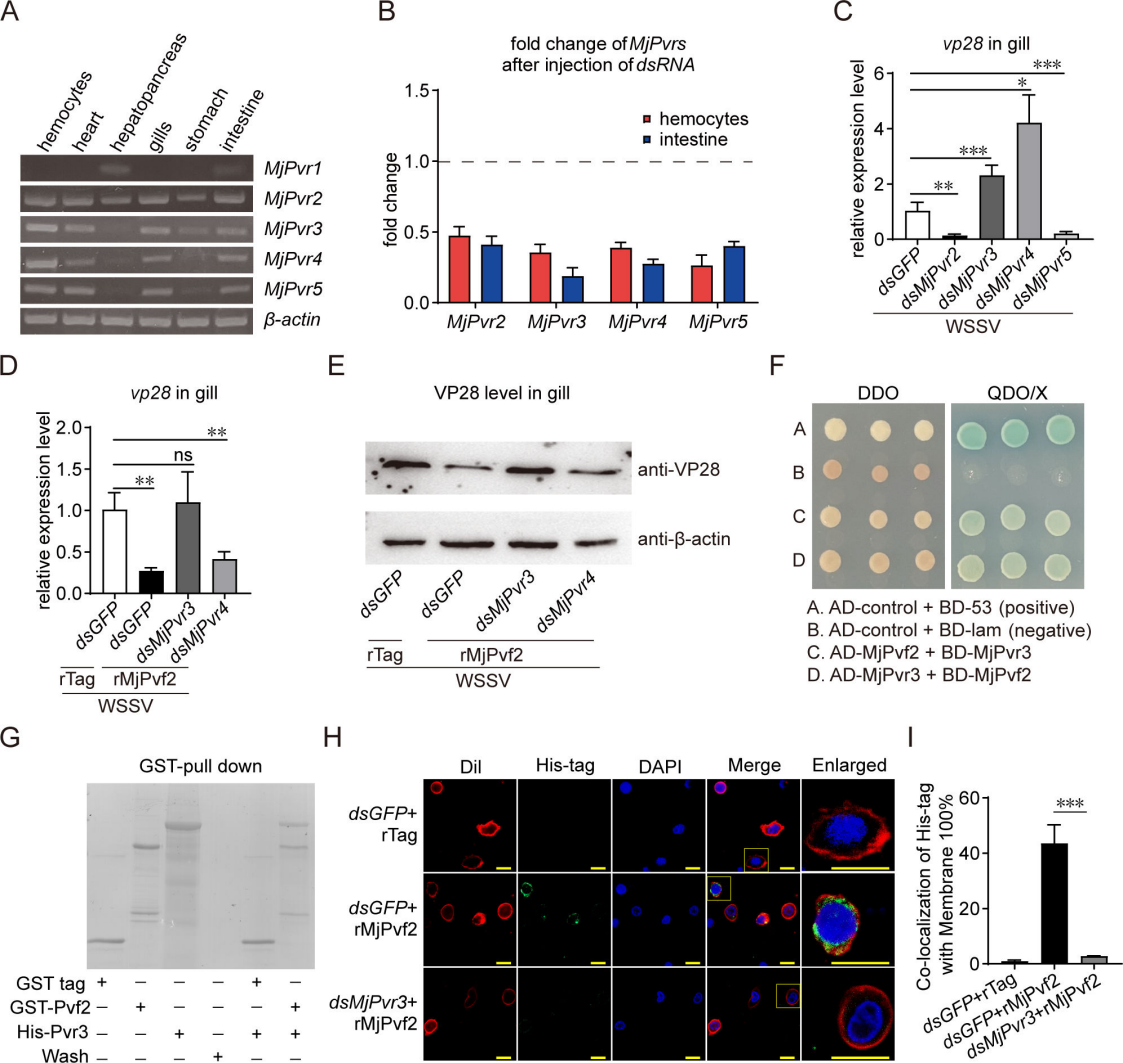
Identification of the downstream receptors of MjPvf2. (**A**) Tissue distribution of *MjPvrs* mRNA. Total RNA was extracted from healthy shrimp. Gene expression was detected using RT-PCR with *β-actin* as an internal reference. Each set of samples contained at least five shrimp. Data represent the results of two independent experiments. (**B**) RNAi efficiency of *MjPvrs*. Total RNA was extracted from hemocytes and intestine 24 h after dsRNA injection. qRT-PCR was used to detect RNAi efficiency. (**C**) WSSV *vp28* transcript levels after *MjPvrs* knockdown. Shrimp were infected with WSSV 24 h after dsRNA injection. Total RNA in gill was then extracted and quantified by qRT-PCR 24 h after WSSV infection. (**D-E**) Effect of *MjPvrs* knockdown on MjPvf2-inhibited WSSV infection. Injection of rMjPvf2 or rTag (3 µg) and WSSV was performed 24 h after dsRNA injection. The WSSV *vp28* transcript levels (**D**) and VP28 translation levels (**E**) in gill were detected 24 h later. (**F**) Interaction of MjPvf2 with MjPvr3. Fragments of the MjPvf2 mature peptide and MjPvr3 extracellular domain were ligated to pGADT7 and pGBKT7 plasmids, respectively. The two treated assay plasmids were transformed into Y2H yeast cells and screened for detection using DDO (Leu-/Trp-) and QDO/X (Ade-/Leu-/Trp-/His-/X-gal+) media. AD, activating domain; BD, binding domain. (**G**) The binding between Pvf2 and Pvr3 was revealed by pull-down assays. GST-Pvf2 or GST-tagged control (GST-tag) was, respectively, mixed and incubated with His-Pvr3. Next, 100 μL of GST resin was added to the mixture to pull down the interacting proteins. The final wash solution and elution solution were collected and analyzed by SDS-PAGE with Coomassie Brilliant Blue staining. (**H**) Immunofluorescence assay of rMjPvf2 deposition on the surface of hemocytes. Scale bar = 5 µm. The images are representative of three independent replicates. (**I**) Quantitative analysis of MjPvf2 on the surface of hemocytes according to three independent replicates using the ImageJ software. Data are the mean ± SD of three independent replicates. Statistical analysis was performed using the Student’s *t-test*. *P < 0.05, **P < 0.01, ***P < 0.001, and ns, not significant. All samples were obtained from at least five shrimp, and the experiment was repeated three times.

Next, we explored the mechanism by which MjPvf2 inhibits viral infection. MjPvf2 belongs to a class of cytokines that are secreted extracellularly and subsequently bind to cell-surface receptors to initiate downstream signaling. To clarify the receptor involved in the antiviral activity of MjPvf2, the potential participation of MjPvr family members was examined following WSSV infection. Tissue distribution showed that all members except *MjPvr1* were expressed in hemocytes and intestine (Fig. 3A). RNAi was subsequently performed to screen for receptors exhibiting effects similar to those observed for MjPvf2. Injection of certain dsRNA significantly inhibited the expression of corresponding *MjPvr*s (Fig. 3B). After WSSV infection, *vp28* expression was analyzed in dsRNA-treated shrimp. As shown in Fig. 3C, knockdown of either *MjPvr3* or *MjPvr4* resulted in increased *vp28* transcription, resembling the effect observed upon MjPvf2 silencing. To examine whether *MjPvr3* or *MjPvr4* is required for the antiviral role of MjPvf2, *MjPvr3* or *MjPvr4* was pre-silenced prior to rMjPvf2 treatment and WSSV infection. In *dsGFP*-treated shrimp, administration of rMjPvf2 reduced *vp28* transcription and VP28 protein level. Silencing of *MjPvr3* abolished the antiviral effect of rMjPvf2, whereas silencing of *MjPvr4* did not abolish this effect (Fig. 3D and E). This suggested that the antiviral function of MjPvf2 might depend on MjPvr3.

To further verify whether MjPvf2 interacts with MjPvr3, yeast two-hybridization and pull-down assays were performed. These assays showed that MjPvf2 interacts with the extracellular ligand-binding domain of MjPvr3 (Fig. 3F and G). In addition, immunofluorescence analysis showed that rMjPvf2 localized to the plasma membrane, whereas this attachment was significantly inhibited after *MjPvr3* knockdown (Fig. 3H and I). These results suggested that MjPvr3 is a receptor for the antiviral function of MjPvf2.

### MjPvf2/MjPvr3 signaling inhibits WSSV infection by relieving lipid consumption

**Fig 4:**
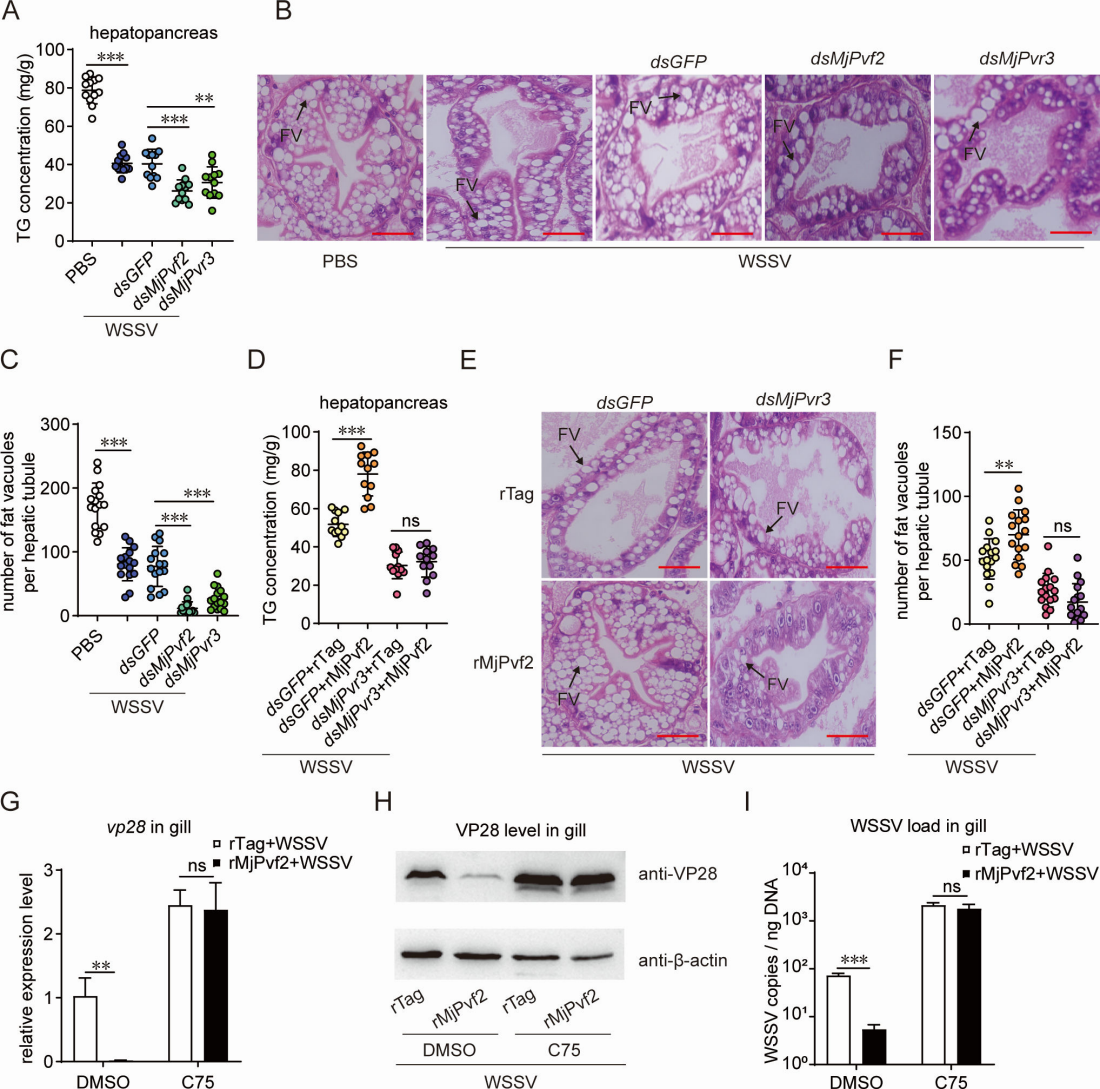
Effect of MjPvf2/MjPvr3 on lipids. (**A**) Effect of *MjPvf2* and *MjPvr3* RNAi on TG content in shrimp. Shrimp were infected with WSSV 24 h after dsRNA injection. TG content in the hepatopancreas was measured using the kit 12 h post-infection. One point represents one shrimp. Data represent two independent replicates. (**B and C**) Effect of *MjPvf2* and *MjPvr3* RNAi on shrimp hepatopancreas. Shrimp were infected with WSSV 24 h after dsRNA injection. The hepatopancreas was collected 12 h after infection for tissue sectioning and H&E staining (**B**). The histomorphology and number of fat vacuoles (FV) in the hepatopancreas were observed. Black arrows show the FV. Scale bar = 20 µm. Data represent two independent replicates. Statistical analysis of the number of FV (**C**). Data are the results of 16 random fields of view. (**D**) Inhibition of the effect of rMjPvf2 on TG content by *MjPvr3* knockdown. Shrimp were infected with WSSV 24 h after dsRNA injection. rMjPvf2 or rTag (3 µg) was injected simultaneously with the WSSV infection. After 12 h, the TG content in the hepatopancreas was measured using a kit. One point represents one shrimp. Data represent two independent replicates. (**E and F**) Inhibition of the effect of rMjPvf2 on shrimp hepatopancreas by *MjPvr3* knockdown. Shrimp were infected with WSSV 24 h after dsRNA injection. rMjPvf2 or rTag (3 µg) was injected simultaneously with the WSSV infection. The hepatopancreas was collected 12 h after infection for tissue sectioning and H&E staining (**E**). Black arrows show the FV. Scale bar = 20 µm. Data represent two independent replicates. Statistical analysis of the number of FV (**F**). Data are the results of 16 random fields of view. (**G–I**) Effect of C75 on MjPvf2-inhibited WSSV infection; 30 µL C75 (1 µg/µL) was injected into shrimp, with DMSO as a control. After 12 h, the shrimp were injected with WSSV (5 × 10^5^ copies) and rMjPvf2 (3 µg), and the control group was injected with the same dose of rTag. After 24 h, WSSV *vp28* transcript level (**G**) and viral load (**I**) in gill were detected using qRT-PCR. WSSV VP28 translation level was detected by western blotting, using β-actin as an internal reference (**H**). Data are the mean ± SD of three independent replicates. Statistical analysis was performed using the Student’s *t-*test. **P < 0.01, ***P < 0.001, and ns, not significant.

Previous studies have reported that Pvf signaling participates in lipid metabolic regulation in *Drosophila* (7), and lipid metabolism has also been implicated in WSSV infection (22). Based on these observations, we examined whether MjPvf2/MjPvr3 signaling influences viral infection through modulation of host lipid metabolism. As the hepatopancreas serves as a key lipid mobilization organ, the expression of *MjPvf2* and *MjPvr3* was upregulated in this tissue following WSSV infection (Fig. S1). Next, we examined the effects of MjPvf2/MjPvr3 on lipid metabolism. As shown in Fig. 4A, WSSV infection led to a significant decrease in TG content in the shrimp hepatopancreas. This reduction was further exacerbated by the knockdown of either *MjPvf2* or *MjPvr3*. To morphologically assess lipid alterations in the shrimp hepatopancreas during WSSV infection, histological analysis was performed using hematoxylin and eosin (H&E) staining. Under normal conditions, hepatic tubules in the hepatopancreas were well organized and contained abundant fat vacuoles. In contrast, WSSV infection disrupted normal hepatic tubule morphology and markedly reduced the number of fat vacuoles (Fig. 4B and C). These pathological changes were more pronounced following *MjPvf2* or *MjPvr3* knockdown, suggesting enhanced lipid consumption during WSSV infection.

To further determine the effects of MjPvf2/MjPvr3 signaling in lipid regulation, *MjPvr3* was first knocked down, followed by the administration of rMjPvf2. Injection of rMjPvf2 alleviated the WSSV-induced decrease in TG levels, whereas this effect was compromised after *MjPvr3* knockdown (Fig. 4D). Consistently, rMjPvf2 protected the morphological integrity of hepatic tubules in the hepatopancreas and increased the number of fat vacuoles, while *MjPvr3* knockdown diminished these protective effects (Fig. 4E and F). These results suggested that MjPvf2/MjPvr3 relieve virus-induced lipid consumption and protect tissues from damage during WSSV infection.

To clarify whether lipid accumulation contributes to the antiviral effect of MjPvf2, lipid synthesis was blocked using the fatty acid synthase inhibitor C75 prior to rMjPvf2 administration in WSSV-infected shrimp. Inhibition of lipid synthesis attenuated the suppressive effects of rMjPvf2 on *vp28* transcription (Fig. 4G) and VP28 translation (Fig. 4H), and viral load (Fig. 4I). This indicated that inhibition of lipid synthesis affects the antiviral function of MjPvf2. Taken together, these results suggested that MjPvf2/MjPvr3 signaling inhibits viral infection by relieving virus-induced lipid consumption.

### MjPvf2/MjPvr3 signaling raises lipid levels by inducing hydroxysteroid dehydrogenase (MjHSD) expression

**Fig 5:**
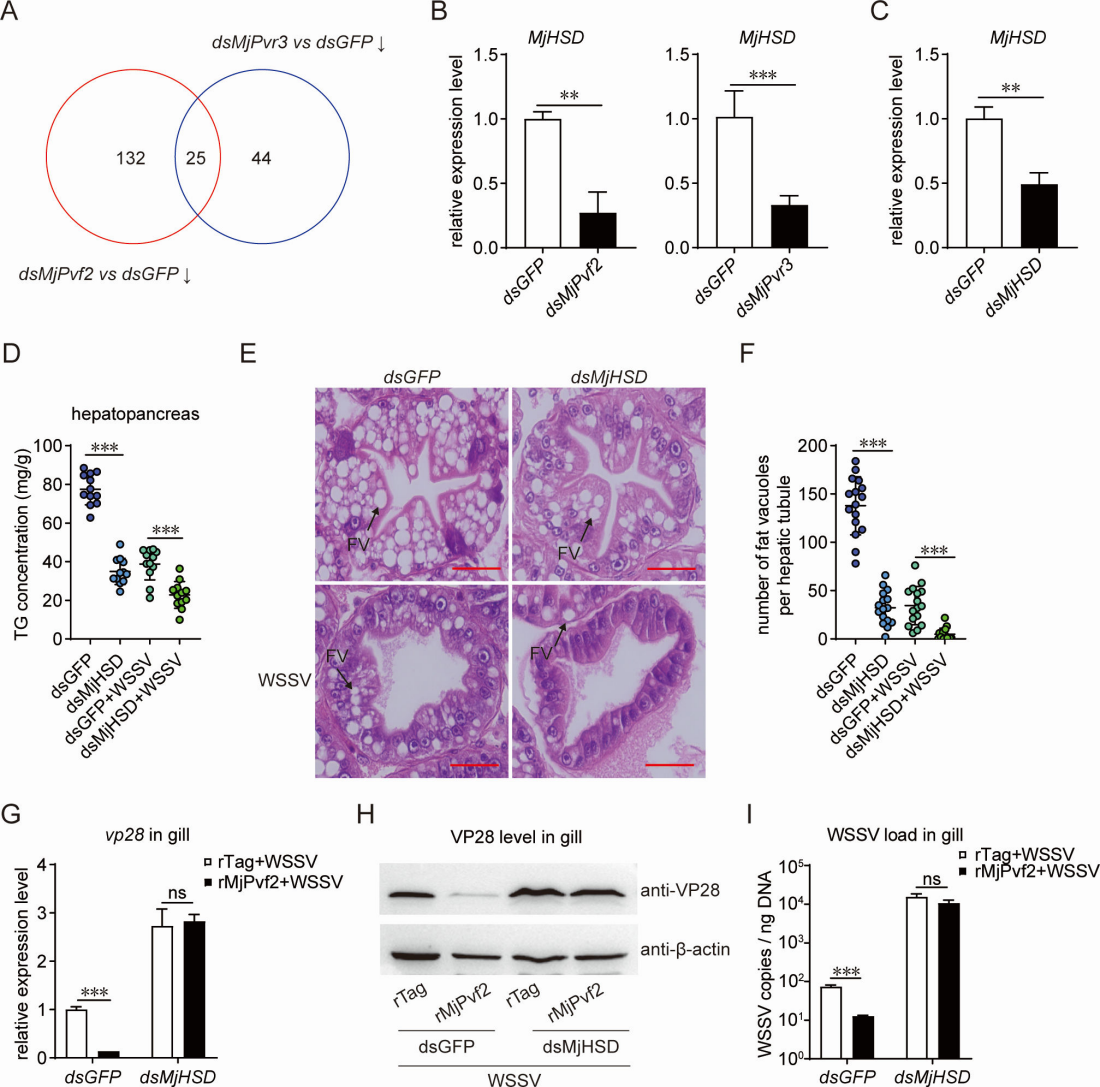
Identification of downstream target genes of MjPvf2/MjPvr3. (**A**) Screening for differentially expressed genes using transcriptome sequencing after *MjPvf2* and *MjPvr3* knockdown. Each group contained three parallel replicates. The selection parameters were FPKM ≥ 2, FC ≥ 2, and FDR ≤ 0.05. (**B**) Validation of transcriptome sequencing results. The expression of *MjHSD* was verified using qRT-PCR after *MjPvf2* and *MjPvr3* knockdown. Data are the mean ± SD of three independent replicates. (**C**) RNAi efficiency of *MjHSD*. Total RNA was extracted 24 h after dsRNA injection. qRT-PCR was used to detect RNAi efficiency. Data are the mean ± SD of three independent replicates. (**D**) Effect of TG content in shrimp after *MjHSD* knockdown. Shrimp were infected with WSSV 24 h after dsRNA injection. TG content in the hepatopancreas was measured 12 h post-infection. One point represents one shrimp. Data represent two independent replicates. (**E-F**) Effect of *MjHSD* RNAi on shrimp hepatopancreas. Shrimp were infected with WSSV 24 h after dsRNA injection. The hepatopancreas was collected 12 h after infection for tissue sectioning and H&E staining (**E**). The histomorphology and number of FV in the hepatopancreas were observed. Black arrows show the FV. Scale bar = 20 µm. Data represent two independent replicates. (**F**) Statistical analysis of the number of FV. Data are the results of 16 random fields of view. (**G–I**) Effect of *MjHSD* RNAi on MjPvf2-inhibited WSSV infection. Shrimp were injected with WSSV 24 h after dsRNA injection, along with rMjPvf2 or rTag (3 µg). After 24 h, WSSV *vp28* transcript level (**G**) and viral load (**I**) in gill were detected using qRT-PCR. WSSV VP28 translation level was detected by western blotting, using β-actin as an internal reference (**H**). Data are the mean ± SD of three independent replicates. Statistical analysis was performed using the Student’s *t-*test. **P < 0.01, ***P < 0.001, and ns, not significant.

To explore how MjPvf2/MjPvr3 signaling regulates lipid levels, downstream target genes were screened using transcriptome analysis following *MjPvf2* and *MjPvr3* knockdown. Twenty-five candidate genes were identified that were consistently downregulated under both conditions (Fig. 5A). Among these candidates, we focused on *MjHSD*, a gene associated with lipid metabolism, as its homolog in mice has been reported to enhance hepatic adipogenesis (23). The sequencing results were validated using qPCR, which showed that the *MjHSD* expression was significantly reduced upon inhibition of *MjPvf2* or *MjPvr3* (Fig. 5B). To determine the effect of MjHSD on lipid metabolism, RNAi was performed to silence *MjHSD* (Fig. 5C). Suppression of *MjHSD* resulted in a significant decrease in TG content in hepatopancreas (Fig. 5D). Histological analysis showed that although the overall hepatic tubule structure remained intact, the number of lipid vacuoles was significantly reduced following *MjHSD* knockdown (Fig. 5E and F). These results suggested that MjHSD, a target gene downstream of MjPvf2/MjPvr3, is involved in regulating lipid levels in shrimp.

To further investigate the contribution of MjHSD to the antiviral function of MjPvf2, rMjPvf2 was administered after *MjHSD* knockdown, followed by WSSV infection. After *MjHSD* inhibition, the WSSV *vp28* transcription level (Fig. 5G) and VP28 translation level (Fig. 5H) significantly increased, as well as the WSSV viral load in the tissues (Fig. 5I). Under these conditions, no significant difference in viral infection levels was observed between rMjPvf2- and rTag-treated shrimp. These results suggested that MjHSD functions downstream of MjPvf2/MjPvr3 signaling to regulate lipid levels and contributes to the antiviral effects of MjPvf2 during WSSV infection.

### MjPvf2 induces MjHSD expression by enhancing SREBP transcriptional activity

**Fig 6:**
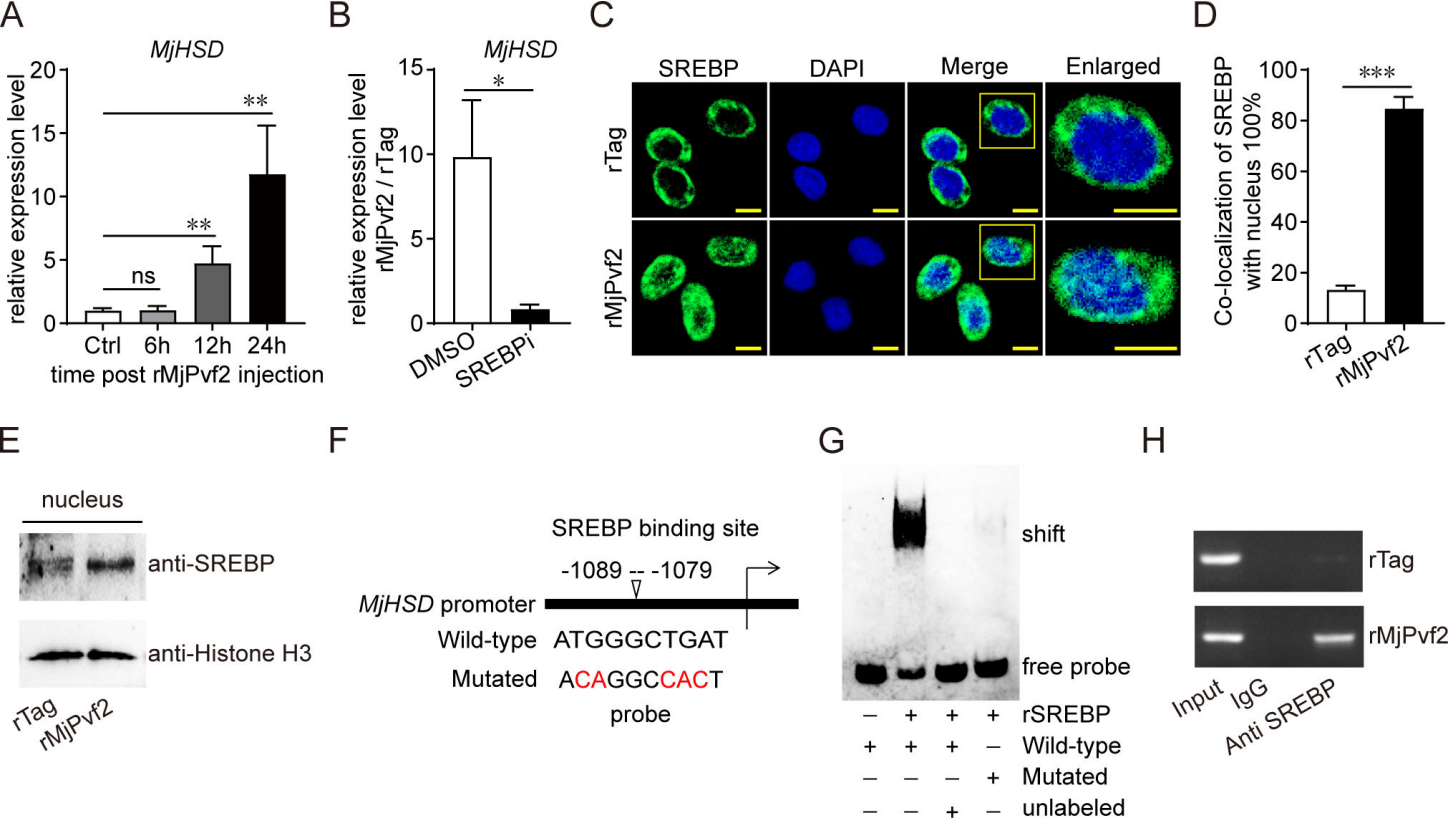
MjPvf2 regulates *MjHSD* expression by enhancing SREBP transcriptional activity. (**A**) *MjHSD* expression pattern. Total RNA was extracted at different time points after injection of rMjPvf2 (3 µg). qRT-PCR was used to detect *MjHSD* expression. Data are the mean ± SD of three independent replicates. (**B**) Effect of SREBP inhibitors on *MjPvf2* regulation of *MjHSD*. Three hours after injection of SREBP inhibitors (5 µg), rMjPvf2 or rTag (3 µg) was injected, and DMSO was used as a control. Total RNA was extracted after 24 h. *MjHSD* expression was detected using qRT-PCR. The FC in *MjHSD* expression is shown as rMjPvf2/rTag. Data are the mean ± SD of three independent replicates. (**C–E**) Induction of SREBP nuclear localization by rMjPvf2. Immunocytochemical analysis of hemocytes 12 h after rTag or rMjPvf2 treatment. Scale bar = 5 µm (**C**). Digitalization of SREBP-nuclei colocalization according to three randomly selected fields using ImageJ software (**D**). Blotting assay of SREBP level in the nucleus 12 h after rTag or rMjPvf2 treatment. Histone H3 was detected as an internal reference for nuclear proteins (**E**). Data represent three independent replicates. (**F**) Promoter analysis of *MjHSD*. The *MjHSD* promoter region was analyzed using the online PROMO 3.0 and JASPAR tools. (**G**) Interaction of rSREBP with biotin-labeled oligonucleotides encoding SREBP-binding sites in the *MjHSD* promoter, as revealed by EMSA. A competitive assay was performed in the presence of excess unlabeled oligonucleotides. Data represent three independent replicates. (**H**) ChIP assay to detect the induction of SREBP-*MjHSD* promoter interactions by rMjPvf2. rMjPvf2 (3 µg) was injected into shrimp, and an equal amount of rTag was used as a control. Hemocytes were collected after 12 h as the pool for the ChIP assay. The immunoprecipitates were analyzed using RT-PCR. Data represent three independent replicates. Statistical analysis was performed using the Student’s *t-*test. *P < 0.05, **P < 0.01, ***P < 0.001, and ns, not significant.

Next, we investigated how MjPvf2 regulates *MjHSD* expression. Treatment with rMjPvf2 significantly increased *MjHSD* expression from 12 to 24 h post-injection (Fig. 6A). Because SREBP is a key transcription factor involved in lipid metabolism (24), we investigated whether SREBP participates in MjPvf2-induced *MjHSD* expression. Pretreatment with an SREBP inhibitor markedly attenuated the induction of *MjHSD* by rMjPvf2 (Fig. 6B). SREBP is a bis-transmembrane protein normally localized to the endoplasmic reticulum membrane. After proteolytic processing and activation, the N-terminal domains of SREBP are released and translocated to the nucleus, where they function as transcription factors (25). To confirm whether MjPvf2 affects SREBP subcellular localization, immunocytochemical analysis was performed after rMjPvf2 injection. As shown in Fig. 6C and D, rMjPvf2 significantly induced mature SREBP into the nucleus. This result was further confirmed by separating and analyzing the nuclear proteins after rMjPvf2 treatment (Fig. 6E). To assess whether SREBP directly regulates MjHSD transcription, the promoter region of MjHSD was analyzed, and a putative SREBP-binding element was identified (Fig. 6F). An electrophoretic mobility shift assay (EMSA) demonstrated that recombinant N-terminal SREBP protein (rSREBP) bound specifically to the wild-type, but not mutated, probes (Fig. 6G). Moreover, chromatin immunoprecipitation (ChIP) assay confirmed the enrichment of *MjHSD* promoter fragments containing SREBP-responsive elements in SREBP immunoprecipitates following rMjPvf2 treatment (Fig. 6H). These data suggested that MjPvf2 promotes *MjHSD* expression by enhancing the transcriptional activity of SREBP.

### MjPvf2 activates SREBP-mediated *MjHSD* transcription by enhancing ERK signaling

**Fig 7:**
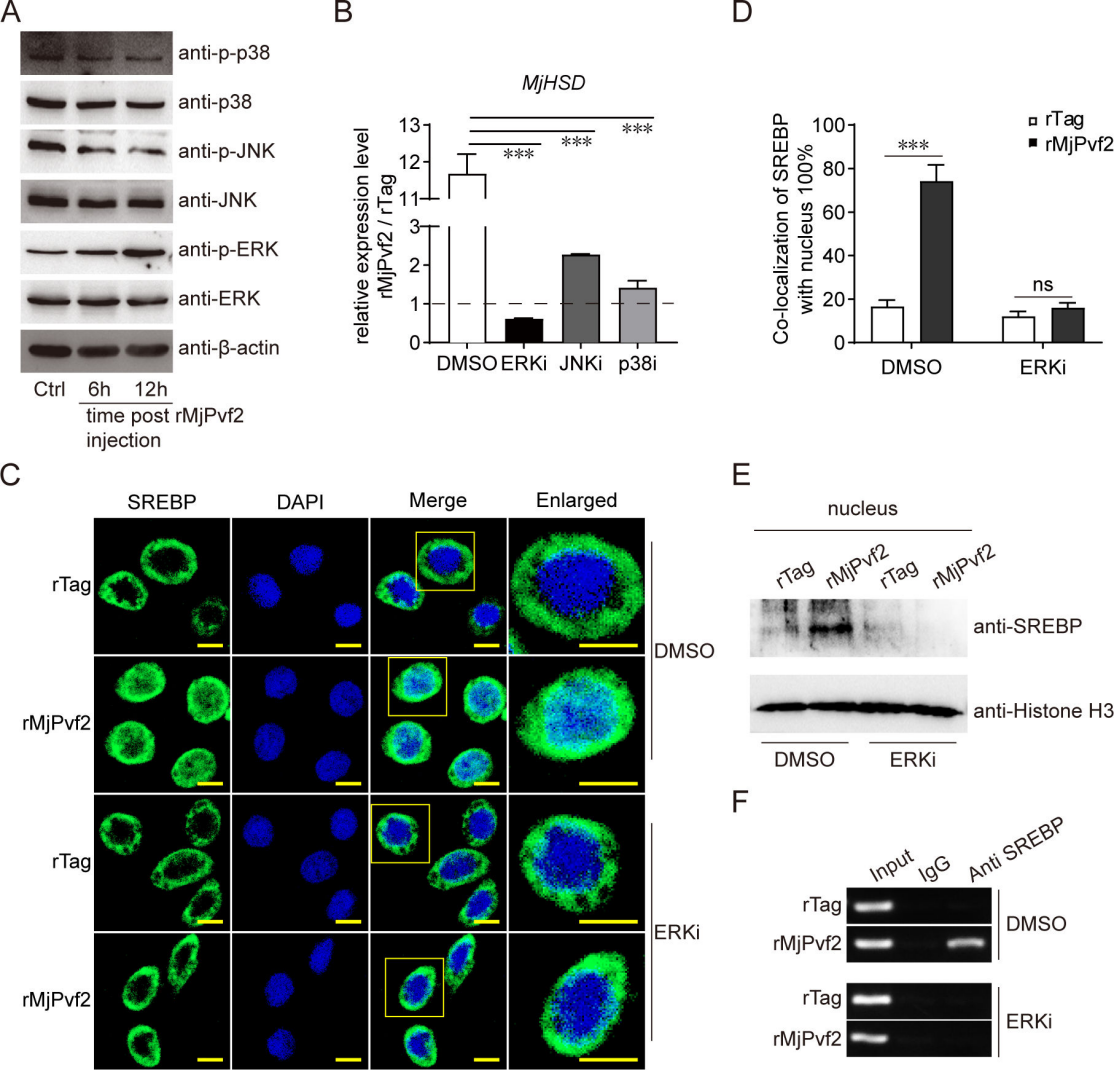
MjPvf2 activates SREBP to induce *MjHSD* transcription by enhancing ERK signaling. (**A**) Effect of MjPvf2 on the MAPK pathway. Total protein was extracted 6 and 12 h after injection of rMjPvf2 (3 µg). Activation of the MAPK pathway was detected using western blotting. (**B**) Effect of MAPK pathway-related inhibitors on MjPvf2-induced *MjHSD* expression. Three hours after injection of each of the three MAPK pathway-related inhibitors (1 µg), rMjPvf2 (3 µg) was injected, and rTag was used as a control. Total RNA was extracted after 24 h. *MjHSD* expression was detected using qRT-PCR. The FC in *MjHSD* expression was shown as rMjPvf2/rTag. (C–E) Inhibition of rMjPvf2-induced SREBP nuclear localization by ERK inhibitor treatment. Three hours after injection of the ERK pathway inhibitor (1 µg), rMjPvf2 (3 µg) was injected, and rTag was used as a control. Immunocytochemical analysis of hemocytes 12 h after rTag or rMjPvf2 treatment. Scale bar = 5 µm (**C**). Digitalization of SREBP-nuclei colocalization according to three randomly selected fields using ImageJ software (**D**). Blotting assay of SREBP level in the nucleus 12 h after rTag or rMjPvf2 treatment. Histone H3 was detected as an internal reference for nuclear proteins (**E**). (**F**) Inhibition of rMjPvf2-induced interaction between SREBP and *MjHSD* promoter by ERK inhibitor treatment. ChIP assay to detect binding of SREBP to the *MjHSD* promoter. Three hours after injection with the ERK pathway inhibitor (1 µg), rMjPvf2 (3 µg) was injected, and rTag was used as a control. Hemocytes were collected after 12 h as the pool for the ChIP assay. The immunoprecipitates were analyzed using RT-PCR. Data are the mean ± SD of three independent replicates. Statistical analysis was performed using the Student’s *t-*test. ***P < 0.001 and ns, not significant. All samples were obtained from at least five shrimp, and the experiment was repeated three times.

Next, we explored the mechanism through which MjPvf2 activates SREBP to regulate *MjHSD* transcription. Mitogen-activated protein kinases (MAPKs) signaling has been reported to regulate lipid metabolism through SREBP (26, 27), and Pvf/Pvr signaling predominantly activates MAPK pathways (28). We therefore assessed the involvement of MAPK signaling in MjPvf2-induced *MjHSD* expression. The blotting analysis showed that ERK phosphorylation, but not p38 and JNK phosphorylation, was significantly enhanced from 6 to 12 h after rMjPvf2 treatment (Fig. 7A). To evaluate the contribution of MAPK pathways to *MjHSD* induction, specific inhibitors targeting ERK, JNK, and p38 were applied prior to rMjPvf2 treatment. Inhibition of each pathway reduced rMjPvf2-induced *MjHSD* expression, with ERK inhibition producing the most pronounced effect (Fig. 7B). This suggested that ERK signaling may play a major role in MjPvf2-induced *MjHSD* expression.

Next, we explored whether ERK affects SREBP activation by MjPvf2. Immunocytochemical analysis showed that rMjPvf2 significantly induces SREBP into the nucleus, whereas this effect was blocked by the ERK inhibitor (Fig. 7C and D). Consistently, isolation of cellular nuclear proteins confirmed that rMjPvf2-induced nuclear enrichment of SREBP was abolished upon ERK pathway inhibition (Fig. 7E). Furthermore, inhibition of the ERK pathway prevented rMjPvf2-induced binding of SREBP to the *MjHSD* promoter (Fig. 7F). These results suggested that MjPvf2 activates SREBP to promote *MjHSD* transcription by enhancing ERK signaling.

## DISCUSSION

By highlighting the role and mechanism of shrimp Pvf/Pvr signaling in antiviral immunity, this study expands current understanding of the functional significance of Pvf/Pvr signaling. In mammals, PDGF/VEGF signaling regulates biological development by controlling cellular proliferation, migration, and survival and by contributing to tissue remodeling, angiogenesis, and hematopoiesis. These signaling pathways are thought to have originated from a common ancestral pathway or to have arisen through convergent evolution (29). PDGF signaling and VEGF signaling are thought to have originated through receptor tyrosine kinases that activate various signaling pathways, including MAPK, JAK/STAT, and PI3K/Akt, thereby regulating diverse physiological processes (30). In *Drosophila*, these factors are uniformly named PDGF/VEGF-related factors (Pvf), and their receptors are named PDGF/VEGF-related receptors (Pvr). The Pvf/Pvr system comprises three ligands (Pvf1-3) and a single receptor (Pvr) and regulates development, immunity, and metabolism, including border cell migration, hemocyte motility and proliferation, antiviral immunity, and lipid storage (5–7, 31–36). The functions of PDGF/VEGF-related proteins have been partially elucidated in a few crustacean species. For example, PVF/PVR signaling affects transglutaminase activity to regulate progenitor cell migration during hematopoiesis in crayfish (9). Additionally, pathogenic infections modulate the expression of these proteins in crustaceans, which may further regulate the host immune response and neuroendocrine system (8, 10). These studies suggested that Pvfs are functionally diverse, but their role in shrimp antiviral immunity has remained unclear. In the present study, we identified six MjPvfs and five MjPvrs in *M. japonicus* and determined the interaction between MjPvf2 and MjPvr3. In addition, we showed that MjPvf2/MjPvr3 signaling exerts an antiviral role during WSSV infection. This study expands current understanding of Pvf/Pvr signaling in immune regulation and demonstrates the functional diversity of the Pvfs.

The current findings further highlight the involvement of lipid metabolism in host-virus interactions. Lipids are closely associated with viral pathogenesis and host antiviral response. During viral invasion, lipids can function as viral receptors or cofactors that aid viral entry into host cells (37–40), and viruses may also hijack host lipid metabolism to support viral gene expression, particle assembly, and maturation (41, 42). Conversely, the host can inhibit viral infection by regulating lipid metabolism. For example, interferon-inducible transmembrane protein 3 inhibits viral infection by altering intracellular cholesterol homeostasis and preventing viral entry (43). Similar to observations in mammals, the relationship between viruses and lipid metabolism in shrimp is complicated. Our previous study showed that Mindin promotes WSSV infection by activating lipophagy and consuming lipids, leading to a reduction in TG content in *M. japonicus* (21). In contrast, the present study demonstrates that the antiviral capacity of MjPvf2/MjPvr3 signaling is correlated with the relieving of virus-induced lipid depletion during WSSV infection and that inhibition of lipid synthesis disrupted this antiviral function. Although lipid synthesis is closely related to MjPvf2/MjPvr3-mediated antiviral immunity, this study investigated the lipid metabolism at the level of overall lipid content; the specific types of lipids regulated by the current mechanism need further elucidation.

This study also revealed a new mechanism by which virus infection affects shrimp lipid metabolism. Lipid metabolism is an intricate biochemical process that relies on multiple enzymes to maintain lipid homeostasis. The HSD family constitutes key enzymes involved in lipid metabolism (44). Here, we show that MjHSD, the HSD member in *M. japonicus*, is regulated by MjPvf2/MjPvr3 signaling and increases TG content (Fig. 5D). Furthermore, SREBP, a pivotal transcription factor in lipid metabolism, was involved in MjPvf2/MjPvr3-mediated regulation of *MjHSD* expression. This finding is similar to a previous study in which SREBP was identified as a direct regulator of 17β-HSD13 in murine models (45). We further demonstrated that MjPvf2/MjPvr3 signaling activates SREBP through enhanced ERK signaling, thereby regulating *MjHSD* expression and lipid levels in shrimp. Therefore, these results revealed an interrelationship between WSSV and host lipid metabolism: WSSV infection modulates host lipid metabolism for successful infection and replication, while the resulting activation of host lipid regulatory pathways induces lipid-associated factors that alleviate lipid depletion and restrict viral infection. The various components of this mechanism provide candidate targets for developing viral disease prevention and control strategies in shrimp aquaculture.

Although Pvf/Pvr signaling has been implicated in either promoting or inhibiting lipid metabolism in various biological processes, this study emphasizes its functional relevance in the context of viral infection. In the cancer model, the expression of PDGFC is upregulated, through interaction with PDGFR, and regulates fatty acid‐associated lipid metabolism; enhanced fatty acid accumulation driven by this pathway accelerates the malignant progression of pancreatic ductal adenocarcinoma (PDAC) (46). Differently, *Drosophila* muscle-derived Pvf1 activates the PI3K/Akt1/TOR signaling in oenocytes to suppress lipid synthesis in hepatocyte-like cells, thereby limiting adipose lipid expansion and maintaining systemic lipid homeostasis (47). In the current context of shrimp-virus interaction, WSSV infection enhances the MjPvf2/MjPvr3 signal, thereby activating ERK/SREBP to promote lipid synthesis. This regulation is an antiviral strategy of the host to defend against viral infections. Therefore, this study suggests that the modulation of lipid metabolism by Pvf/Pvr signaling is enhanced not only in developmental processes but also under diseased circumstances. In the shrimp-WSSV interaction context, this enhancement helps the shrimp host to cope with the WSSV infection-caused disruption of lipid metabolism, restore normal lipid levels, and resist WSSV infection.

In summary, we identified an MjPvf2/MjPvr3/ERK/SREBP/MjHSD signaling pathway in WSSV-host lipid metabolism interactions, which can exert antiviral functions by promoting lipid synthesis and alleviating WSSV-caused lipid depletion in shrimp. This not only reveals the role of the Pvf/Pvr system in shrimp antiviral immunity but also provides new insights for establishing immune control strategies for shrimp diseases and understanding virus-host interactions.

## MATERIALS AND METHODS

### Animals and viral inoculum preparation

Healthy kuruma shrimp (*M. japonicus*, 5–8 g) were purchased from a farm in Jimo, Shandong, China, and temporarily cultured in oxygenated seawater at 25 °C, and fed commercial diets daily in the laboratory. Randomly selected individuals were screened using established shrimp-specific pathogen detection methodologies to verify the absence of targeted pathogens. Subsequently, pathogen-free shrimp were randomly selected for the experiments.

The original WSSV inoculum used in this study was gifted from the East China Sea Fishery Institute, Shanghai, China. To prepare successive inocula, the original inoculum was injected into red swamp crayfish intramuscularly. The gills of moribund crayfish were collected and homogenized in phosphate-buffered saline (PBS) (140 mM NaCl, 2.7 mM KCl, 10 mM Na_2_HPO_4_, 1.8 mM KH_2_PO_4_, pH 7.4) at a ratio of 1:10 (wt/vol). The homogenate was repeatedly frozen and thawed and centrifuged at 3,000 × *g* for 10 min at 4°C. The supernatant was filtered through a 0.45 μm filter. The obtained filtrate was diluted to the appropriate titer with PBS and used as the viral inoculum. Shrimp were injected with the WSSV inoculum (5 × 10^5^ virions/shrimp). Equal amounts of PBS were used as controls.

### Post-treatment sample collection

Total RNA was extracted using TRIzol Reagent (15596-026; Invitrogen, Carlsbad, CA, USA). Hemolymph was collected in pre-chilled anticoagulant (0.45 M NaCl, 10 mM KCl, 10 mM EDTA, and 10 mM HEPES, pH 7.45), centrifuged at 800 × *g* for 8 min at 4°C, and the collected hemocytes were suspended in TRIzol. The remaining tissues were ground using TRIzol in a homogenizer. The extracted total RNA was solubilized in DEPC water, and cDNA synthesis was performed using a ReverTra Ace qPCR RT Kit (FSQ-101; Toyobo, Osaka, Japan) according to the manufacturer’s instructions. The synthesized cDNA was stored at −20°C for subsequent qRT-PCR. Genomic DNA was extracted using the MagExtractor Genome (NPK-101; Toyobo). The WSSV copy number was measured as previously described (48). Protein samples were extracted by homogenizing the tissues using Radioimmunoprecipitation Assay Lysis Buffer (P0013B; Beyotime, Wuhan, China). The tissue homogenate was centrifuged at 12,000 × *g* for 15 min at 4°C. The supernatant was collected and mixed with protein-loading dye (P0015L; Beyotime) and boiled at 100°C for 10 min for subsequent western blotting analysis.

### Quantitative real-time PCR (qPCR)

qPCR was used to detect the changes in gene expression levels. The primers used are listed in Table 1. The system used was the iQ SYBR Green Supermix (170-8882; Bio-Rad, Hercules, CA, USA) and CFX96 Real-time System (Bio-Rad) detection software. The PCR conditions were as follows: 94°C for 10 min, 40 cycles of 94°C for 14 s, and 60°C for 1 min. The melting curve was detected every 1°C from 65 to 95°C. *β-actin* was used as an internal reference. Data were processed using the 2^−ΔΔCT^ method (49).

**TABLE 1:** Primers used in this study

Primer	Sequence (5′−3′)
(q)RT-PCR	
MjPvf1RTF	GCAGTGAGTCGCTGTTCTAT
MjPvf1RTR	TTCAACGGGGATTTCTAAG
MjPvf2RTF	CCCACAGACTCGCTTCCAA
MjPvf2RTR	GTCGGGTCCTCGTCATCCT
MjPvf3RTF	AAGCCCTTGGTGATGTT
MjPvf3RTR	TCGGATTTTGTTGTTGACT
MjPvf4RTF	CTCCTGCTTGTCGCTTCT
MjPvf4RTR	CTTTATCCTTCGCTTGCTG
MjPvf5RTF	GCACCATCCATACCTACCG
MjPvf5RTR	TGTTGTCAAAGGAAGGCTCT
MjPvf6RTF	ACGATGAGGAGTGCCAGTG
MjPvf6RTR	TGAAAGTGTATCCCGAAGAAC
MjPvr1RTF	GACTTTACGCACGACTCTTATG
MjPvr1RTR	GGAGCCTCTGCTGTTTGG
MjPvr2RTF	ATTCAAAATGCGTCCGTA
MjPvr2RTR	CAAAGAGGCTGCCACTATC
MjPvr3RTF	AACAGCGGTGAGTCAAAAC
MjPvr3RTR	AACTGAGACACTGGCGGA
MjPvr4RTF	TACGATAAGCACATCCGACC
MjPvr4RTR	TGGACCACTACGAAGGCG
MjPvr5RTF	GCCACGAGTTCCAGTATGA
MjPvr5RTR	CACAGGTAAGCACCAGAGG
β-actinRTF	CAGCCTTCCTTCCTGGGTATGG
β-actinRTR	GAGGGAGCGAGGGCAGTGATT
VP28RTF	AGCTCCAACACCTCCTCCTTCA
VP28RTR	TTACTCGGTCTCAGTGCCAGA
MjHSDRTF	ACTGTCGTCGGATTCCTTGTTGTG
MjHSDRTR	CGTAAGTCTGAGGCAGTCTCTTGTC
MjStatRTF	GGTCCCAGTTCTGTAAGG
MjStatRTR	TAGGCACATTCGGATAAA
RNAi	
MjPvf2RNAiF	GCGTAATACGACTCACTATAGGCTTCGGATAATGTTCGGTG
MjPvf2RNAiR	GCGTAATACGACTCACTATAGGAGTTGACAGGGACAGGAGC
MjPvr2RNAiF	GCGTAATACGACTCACTATAGGCAGTGGGATTCACGCATAG
MjPvr2RNAiR	GCGTAATACGACTCACTATAGGTGTTTCCTCATTTGGGTTT
MjPvr3RNAiF	GCGTAATACGACTCACTATAGGGATGTGGAAGAACAGCAGG
MjPvr3RNAiR	GCGTAATACGACTCACTATAGGTTATCGGCGTATTTGGGT
MjPvr4RNAiF	GCGTAATACGACTCACTATAGGCCGTGTAAAAGAGTCGCTA
MjPvr4RNAiR	GCGTAATACGACTCACTATAGGTTCTGAATCGGGTATGGTC
MjPvr5RNAiF	GCGTAATACGACTCACTATAGGGTCCTGGGCTTTCCTCTG
MjPvr5RNAiR	GCGTAATACGACTCACTATAGGGTTTCGCCGTTATGCTTG
GFPRNAiF	GCGTAATACGACTCACTATAGGTGGTCCCAATTCTCGTGGAAC
GFPRNAiR	GCGTAATACGACTCACTATAGGCTTGAAGTTGACCTTGATGCC
MjHSDRNAiF	GCGTAATACGACTCACTATAGGAGGCTGTACGAAAGACAAATG
MjHSDRNAiR	GCGTAATACGACTCACTATAGGAAAGGTATCGCTGGTGGG
MjStatRNAiF	GCGTAATACGACTCACTATAGGGACTTTCCTGCTCCGTTTC
MjStatRNAiR	GCGTAATACGACTCACTATAGGGCGTTGGCACTGTTGAGAC
Recombinant expression	
MjPvf2F	GCCATGGCTGATATCGGATCCATGGTGCGCTTGGCTTCG
MjPvf2R	GTGGTGGTGGTGGTGCTCGAGTTACCTCCGGCTGACGGTG
MjSREBPF	GATCTGGTTCCGCGTGGATCCCAGGGAGGCCTGGGAGGC
MjSREBPR	GTCACGATGCGGCCGCTCGAGGCGTGAGCCGTCCGCCAT
GST-Pvf2F	GATCTGGTTCCGCGTGGATCCATGGTGCGCTTGGCTTCG
GST-Pvf2R	GTCACGATGCGGCCGCTCGAGTTACCTCCGGCTGACGGTG
His-Pvr3F	GCCATGGCTGATATCGGATCCGAACCTACAATAGCATCTAACAAGAGG
His-Pvr3R	GTGGTGGTGGTGGTGCTCGAGTTAGGGTCATCTTTGTCTCCTTGA
Yeast two-hybrid assay	
MjPvf2-AD-F	GCCATGGAGGCCAGTGAATTCATGGTGCGCTTGGCTTCG
MjPvf2-AD-R	CAGCTCGAGCTCGATGGATCCCCTCCGGCTGACGGTGTAA
MjPvf2-BD-F	ATGGCCATGGAGGCCGAATTCATGGTGCGCTTGGCTTCG
MjPvf2-BD-R	CCGCTGCAGGTCGACGGATCCCCTCCGGCTGACGGTGTAA
MjPvr3-AD-F	GCCATGGAGGCCAGTGAATTCGAACCTACAATAGCATCTAACAAGAGG
MjPvr3-AD-R	CAGCTCGAGCTCGATGGATCCTAGGGTCATCTTTGTCTCCTTGATC
MjPvr3-BD-F	ATGGCCATGGAGGCCGAATTCGAACCTACAATAGCATCTAACAAGAGG
MjPvr3-BD-R	CCGCTGCAGGTCGACGGATCCTAGGGTCATCTTTGTCTCCTTGATC
ChIP	
MjHSD-chip-F	CATCTTGCACATTACGATTTT
MjHSD-chip-R	AGAGCACGATGATTTTGATT

### RNA interference

Primers incorporating the T7 promoter sequence were designed and synthesized for the target gene. Specific primers were used to amplify DNA fragments as templates for dsRNA synthesis (Table 1). The corresponding dsRNA was synthesized using a T7 RNAi Transcription Kit (TR102; Vazyme, Nanjing, China). dsRNA for green fluorescent protein was used as a control. The corresponding gene expression was knocked down by injecting 5 µg/g into the shrimp abdomen using a microinjector. RNAi efficiency was assayed at 24 h and 48 h after injection.

### Recombinant protein expression and purification

The sequence encoding the MjPvf2 (XM_043032460.1) maturation peptide was amplified using primers listed in Table 1, and ligated into the pET-30a (+) plasmid and pGEX-4T-2 plasmid. The sequence encoding the SREBP (XM_043038038.1) N-terminal domain was amplified using primers listed in Table 1 and ligated into the pGEX-4T-2 plasmid. The sequence encoding the MjPvr3 (XM_043025242.1) extracellular domain was amplified using primers listed in Table 1 and ligated into the pET-32a (+) plasmid. The recombinant product was transformed into *Escherichia coli* Rosetta (DE3), and positive clones were selected. Expression was induced with 0.2 mM isopropyl β-D-thiogalactopyranoside (IPTG) at 28°C for 7 h. rMjPvf2 was expressed in inclusion bodies. The inclusion bodies were washed and dissolved in buffer (0.1 mM Tris-HCl, pH 8.0, 10 mM DTT, 8 M Urea), dialyzed with PBS buffer containing 5% glycerol for renaturation, and used within 2 days. An empty vector was used to prepare a tag protein (rTag) as a control. Recombinant proteins (3 µg) were injected into the shrimp. rSREBP and GST-Pvf2 were expressed as a soluble protein. ProteinIso GST resin (DP-201; TransGen Biotech, Beijing, China) was used as the affinity matrix, and elution was achieved by adding glutathione. rSREBP was dialyzed with PBS buffer containing 5% glycerol within 2 days. All the proteins were stored at −80°C for subsequent experiments.

### Antibody preparation

The rMjPvf2 solution (1 mg/mL, 1.5 mL) and an equal volume of complete Freund’s adjuvant (F5881; Sigma-Aldrich, St. Louis, MO, USA) were completely emulsified. This mixture was subcutaneously injected into New Zealand white rabbits. A second immunization was performed 24 days later, and the complete adjuvant was replaced with an incomplete adjuvant (F5506; Sigma-Aldrich). Rabbit antiserum was collected after 7 days and stored at −80°C for subsequent experiments.

### Western blotting

Protein expression levels were determined using western blotting. Equal amounts of total protein (100 µg per lane) were separated using 12.5% sodium dodecyl sulfate polyacrylamide gel electrophoresis. Proteins were then transferred onto nitrocellulose membranes using a semi-dry membrane transfer machine (Jim-X, Dalian, China). The membranes were blocked with 5% skim milk in Tris-buffered saline (TBS) for 45 min and then incubated with specific primary antibodies for 3 h at room temperature or overnight at 4°C. The membranes were subsequently washed three times with TBST (TBS with Tween-20) and once with TBS before incubation with secondary antibodies for 3 h at room temperature. Immunoreactive protein bands were imaged and photographed using a High-sig ECL Immunoblot Color Developer (180-5001; Tanon, Shanghai, China) and Tanon 5200 Chemiluminescence Imaging System.

For the detection of target proteins and β-actin, the membrane was cut after transfer according to the molecular weight of the target protein, and the corresponding membrane pieces were incubated separately with the indicated primary antibodies. The primary antibodies used were anti-MjPvf2 (1:100 dilution), anti-β-actin (1:2,000 dilution), anti-VP28 (1:300 dilution), anti-ERK (1:200 dilution), anti-JNK (1:200 dilution), and anti-p38 (1:200 dilution). Commercial primary antibodies included anti-p-ERK (AP0472; ABclonal, Wuhan, China), anti-p-JNK (AP0473; ABclonal), anti-p-p38 (AP0526; ABclonal), and anti-SREBP (WL02093; Wanleibio, Shenyang, China), all used at a 1:1,000 dilution. The secondary antibody used was goat anti-rabbit antibody coupled with horseradish peroxidase (ZB-2301; ZhongShan Bio-Tech, Beijing, China; 1:10,000 dilution).

### Yeast two-hybrid experiment

The experiments were performed using the Matchmaker Gold Yeast Two-Hybrid System (630495; Clontech, USA). Fragments encoding the MjPvf2 maturation peptide and MjPvr3 extracellular domain were amplified using primers listed in Table 1 and ligated to the pGADT7 and pGBKT7 plasmids, respectively. The two pairs of plasmids to be tested for interactions were transformed into Y2H yeast cells and cultured in DDO (SD/-Leu/-Trp) medium for screening. The screened positive clone strains were inoculated onto QDO/X (SD/-Ade/-Leu/-Trp/-His/X-α-gal) medium for further screening. Strains that grew on DDO medium and grew as blue spots on QDO/X medium indicated the presence of interactions.

### Pull-down assay

A pull-down assay was performed to ascertain the interaction between Pvf2 and Pvr3. The recombinant GST-Pvf2 and His-Pvr3 proteins were expressed and purified according to the following established protocols for recombinant protein expression and purification. The recombinant GST-Pvf2 or GST-tagged control proteins (as bait) were individually mixed with His-Pvr3 in binding buffer, with each protein used at 100 μg. After incubation at 4°C for 6 h with gentle rotation, 100 μL of GST resin (C600031; BBI, Shanghai, China) was added to the mixture, which was subsequently incubated at 4°C for 45 min. Next, the mixture was centrifuged at 600 × *g* for 2 min to pellet the resin, and the supernatant was discarded. The resin was resuspended in PBS and centrifuged at 600 × *g* for 2 min to remove any residual unbound proteins. After six washes, 50 μL of the elution buffer was added to the resin to elute the interacting proteins. The binding of GST-Pvf2 and His-Pvr3 proteins in the eluate was detected using SDS-PAGE and Coomassie Brilliant Blue staining.

### Deposition of rMjPvf2 on the cell membrane

Detection of rMjPvf2 on cell membranes was performed using immunofluorescence. Hemocytes were collected and placed on slides after injecting *dsGFP* or *dsMjPvr3* into shrimp. Next, rTag or rMjPvf2 (20 μg) was added to slides and incubated for 12 h. After washing three times with PBS, the slides were incubated with His-tag antibodies (TA-02; Zhongshan Biotech; 1:200 dilution in 3% bovine serum albumin [BSA]) overnight at 4°C. Following three washes with PBS, goat anti-mouse IgG DyLight 488 (A23210; Abbkine, Wuhan, China; 1:1,000 dilution in 3% BSA) was added and incubated for 2 h. After washing three times with PBS, hemocytes were stained with the cell membrane red fluorescent probe Dil (C1036; Beyotime; 1:1,000 dilution in PBS) for 15 min at 37°C to stain the cell membrane and DAPI for 10 min to stain the nuclei. Subsequently, the slides were washed with PBS, and images were captured using a Zeiss LSM 900 confocal microscope (Zeiss, Oberkochen, Germany). The images were analyzed using ZEN software (Zeiss).

The colocalization of His-tagged protein and cell membrane was digitized from three randomly selected fields of view, *n* > 100 cells. Image J software was used to analyze the colocalization between Dil (cell membrane) and IgG DyLight 488 (His-tagged protein) fluorescence signal, and rTag was used as the control protein. The number of co-localized cells was calculated. Statistics were defined as (the hemocytes of His-tag-membrane colocalization/all hemocytes observed) × 100%.

### Triglyceride assay

The lipid content in the hepatopancreas of shrimp was measured using a TG Content Assay Kit (BC0620; Solarbio, Beijing, China). One shrimp was used as one sample. The absorbance was measured at 420 nm using a Multiskan FC microplate reader (Thermo Fisher Scientific), and the TG content per gram of hepatopancreatic tissue was calculated.

### Histological analysis

The hepatopancreas of the collected shrimp was fixed using Davidson’s AFA fixative (30% ethanol, 22% formalin, and 11.5% acetic acid). After 24 h of fixation, the tissue was dehydrated, embedded in paraffin, sectioned at 7 μm thickness, and stained with H&E. Sections were observed under a BX51 microscope (Olympus, Tokyo, Japan) and photographed using a DP70 digital camera (Olympus). Hepatopancreatic morphology and the number of fat vacuoles were analyzed.

### Inhibitor treatment

The inhibitors used were C75 (HY-12364) and SREBP inhibitor 125B11 (HY-14452), purchased from MedChemExpress (New Jersey, USA). ERK1/2 inhibitor U0126 (S1102), JNK1/JNK2/JNK3 inhibitor SP600125 (S1460), and p38 MAPK inhibitor SB203580 (S1076), purchased from Selleck Chemicals (Houston, TX, USA). The inhibitors were dissolved in water containing 5% DMSO and 40% PEG300. The inhibitor was injected into shrimp using a microinjector, with the solvent as a control.

Transcriptome analysis *dsGFP*, *dsMjPvf2*, and *dsMjPvr3* were injected into shrimp at 5 µg/g of shrimp (about 5 g) in three groups of at least 30 shrimp each. After 24 h, hemocytes and total intestinal RNA were extracted in triplicate per group. Total RNA was divided into two parts: one for transcriptome analysis and the other for subsequent validation. Transcriptome sequencing was performed using an Illumina platform (San Diego, CA, USA) by Biomarker Technologies, Inc. Bioinformatic analysis was performed using BMKCloud (www.biocloud.net). Gene expression differences were assessed using Fragments Per Kilobase of exon per million mapped fragment (FPKM) values. The parameters analyzed were FPKM ≥ 2, fold change (FC) ≥ 2, and error rate (FDR) ≤ 0.05. The results were visualized using an online Venn diagram tool (bioinformatics.psb.ugent.be/webtools/Venn).

### Analysis of the promoter region

The SMARTer RACE 5′ Kit (634868, Clontech, Mountain View, CA) was first used to clone the 5′-end of cDNA of *MjHSD* according to the manufacturer’s instructions. The transcription start sites of *MjHSD* were identified by integrally comparing and analyzing the cDNA sequences and transcriptome sequencing data sets. The upstream sequences of the transcription start site were obtained by analyzing the *M. japonicus* genome (GenBank GCA_017312705.1) and validated by PCR and sequencing. The analysis of SREBP-binding sites in the promoter of *MjHSD* was performed using the PROMO 3.0 (alggen.lsi.upc.es/cgi-bin/promo_v3) and JASPAR (jaspar.genereg.net) online tools.

### Immunocytochemical analysis

An immunocytochemical assay was performed to detect the subcellular localization of SREBP. Hemolymph was extracted using a cold anticoagulant containing 4% paraformaldehyde for fixing for 10 min and centrifuged at 800 × *g* for 8 min at 4°C to collect the hemocytes. The hemocytes were washed with PBS and spread onto poly L-lysine-coated glass slides. The slides were washed three times with PBS. Next, 0.2% Triton X-100 in PBS was added to the slides and incubated for 10 min. The slides were again washed three times with PBS and blocked with 3% BSA in PBS at 37 °C for 1 h. SREBP antibodies (1:100 diluted in 3% BSA) were added to the slides and incubated overnight at 4 °C. After washing three times with PBS, Goat anti-rabbit Alexa Fluor 488 (A23220, Abbkine; 1:1,000 diluted in 3% BSA) was added and incubated for 2 h in the dark. After washing three times with PBS, DAPI (AS-83210; AnaSpec, San Jose, CA, USA) was added to stain the nuclei for 10 min. Finally, the slides were washed with PBS, and the images were captured using a Zeiss LSM 900 confocal microscope. Images were analyzed using ZEN software.

The colocalization of SREBP and the nucleus was digitized from three randomly selected fields of view, *n* > 100 cells. Image J software was used to analyze the colocalization between DAPI (nucleus) and IgG DyLight 488 (SREBP) fluorescence signal. The number of co-localized cells was calculated. Statistics were defined as (the hemocytes of SREBP-nuclei colocalization/all hemocytes observed) × 100%.

### Separation of nuclear and cytoplasmic proteins

To determine the amount of SREBP in the nucleus, nuclear proteins were extracted using a Nuclear Protein Extraction Kit (R0050; Solarbio) according to the manufacturer’s instructions. Shrimp hemocytes were collected and homogenized using a cytoplasmic protein extraction reagent containing 1 mM phenylmethanesulfonyl fluoride (PMSF) and a phosphatase inhibitor cocktail (M7528; AbMole Bioscience, Houston, TX, USA). The homogenate was shaken for 20 s and placed on ice for 3 min. Following five cycles of alternating treatment, the homogenate was centrifuged at 13,000 × *g* for 20 min at 4°C. The sediment was collected and washed three times with PBS. The sediment was then resuspended in a nucleoprotein extraction reagent containing 1 mM PMSF and a phosphatase inhibitor cocktail, and processed as described above. After centrifugation at 13,000 × *g* for 20 min at 4°C, the supernatant was collected as the nuclear protein. The separated nuclear proteins were quantitatively analyzed using western blotting. At least five shrimp were used for each sample.

### Chromatin immunoprecipitation assay

A ChIP assay was performed using a ChIP assay kit (P2078; Beyotime) to detect the transcriptional regulation of MjHSD by SREBP. Shrimp were injected with rMjPvf2 or rTag (3 µg), and hemocytes were collected 12 h later to serve as the sample pool for ChIP experiments. The immunoprecipitates were analyzed by PCR to determine the presence and amount of specific DNA fragments. The primers used are listed in Table 1.

### Electrophoretic mobility shift assay

EMSA was performed to examine the interaction between SREBP and the *MjHSD* promoter regions. Oligonucleotide probes were synthesized (Sangon Biotech, Shanghai, China) and labeled with biotin using an EMSA Probe Biotin Labeling Kit (GS008; Beyotime). The oligonucleotide sequences are listed in Table 1. EMSA was performed using a Chemiluminescent EMSA Kit (GS009; Beyotime), according to the manufacturer’s instructions. Purified rSREBP (2 μg) was incubated with the probes (5 ng) at 25°C for 20 min in binding buffer. In the competition-binding assays, excess unbiotinylated probes were added to the mixture. The samples were analyzed on 6% acrylamide gels in 0.5 × Tris-borate-EDTA buffer and electroblotted onto a nylon membrane. Labeled DNA was detected and visualized using a chemiluminescent biotin-labeled nucleic acid detection kit.

### Statistical analysis

Data were analyzed using GraphPad Prism 8 software (GraphPad Inc., La Jolla, CA, USA). The remaining data were analyzed using the Student’s *t*-test, and statistical significance was established at *P* < 0.05. *P < 0.05, **P < 0.01, ***P < 0.001, and ns, not significant.

## Data Availability

All data are available in the main text and the supplemental material.

## SUPPLEMENTAL MATERIAL

Fig. S1 jvi.00182-26-s0001.pdf WSSV enhanced the expression of *MjPvf2* and *MjPvr3* in shrimp hepatopancreas.

ASM does not own the copyrights to Supplemental Material that may be linked to, or accessed through, an article. The authors have granted ASM a non-exclusive, world-wide license to publish the Supplemental Material files. Please contact the corresponding author directly for reuse.
