# Supplementary material for: Pvf/Pvr signaling relieves white spot syndrome virus-induced lipid consumption to inhibit viral infection in shrimp
Source: J Virol. 2026 Apr 29;100(5):e00182-26. doi: 10.1128/jvi.00182-26 (PMC13185635; doi:10.1128/jvi.00182-26)
Supplement: Fig. S1 — WSSV enhanced the expression of MjPvf2 and MjPvr3 in shrimp hepatopancreas. [file jvi.00182-26-s0001.pdf]

Supporting figure

S1 Fig

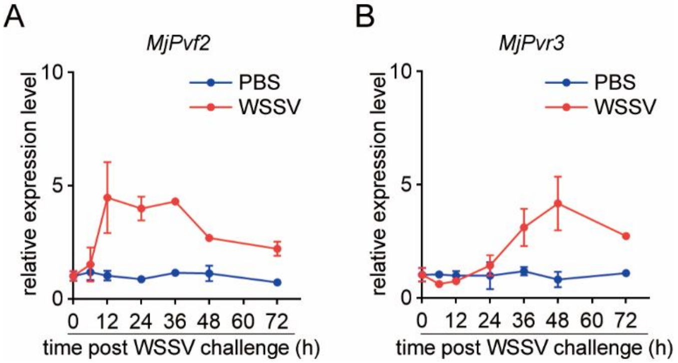

S1 Fig. WSSV enhanced the expression of *MjPvf2* and *MjPvr3* in shrimp hepatopancreas.

(**A-B**) Expression pattern of *MjPvf*2 and *MjPvr3* in shrimp hepatopancreas after WSSV infection. Total RNA was extracted from shrimp hepatopancreas at the corresponding time points after WSSV infection. qRT-PCR was used to detect gene expression, using *β-actin* as an internal reference. Expression levels at each time point are normalized to those of the control group. Results are shown as the mean ± SD. All samples were obtained from at least five shrimp and the experiment was repeated three times.
